# Optimal Processing Parameters of Transmission Parts of a Flapping-Wing Micro-Aerial Vehicle Using Precision Injection Molding

**DOI:** 10.3390/polym14071467

**Published:** 2022-04-04

**Authors:** Huei-Yu Huang, Fang-Yu Fan, Wei-Chun Lin, Chiung-Fang Huang, Yung-Kang Shen, Yi Lin, Muhammad Ruslin

**Affiliations:** 1 Department of Dentistry, Taipei Medical University Shuang Ho Hospital, Taipei Medical University, New Taipei City 23561, Taiwan; dr_hyhuang@tmu.edu.tw; 2 School of Dentistry, College of Oral Medicine, Taipei Medical University, Taipei 11031, Taiwan; 3 School of Dental Technology, College of Oral Medicine, Taipei Medical University, Taipei 11031, Taiwan; fish884027@tmu.edu.tw (F.-Y.F.); weichun1253@tmu.edu.tw (W.-C.L.); chiung0102@tmu.edu.tw (C.-F.H.); 4 Division of Family and Operative Dentistry, Department of Dentistry, Taipei Medical University Hospital, Taipei 11031, Taiwan; 5 Research Center for Biomedical Devices, Taipei Medical University, Taipei 11031, Taiwan; 6 Department of Business Administration, Takming University of Science and Technology, Taipei 114, Taiwan; linyi@takming.edu.tw; 7 Department of Oral and Maxillofacial Surgery, Faculty of Dentistry, Hasanuddin University, Makassar 90245, Indonesia; mruslin@unhas.ac.id

**Keywords:** flapping-wing micro-aerial vehicle, precision injection molding, Taguchi method, warpage, optimization

## Abstract

In this study, we designed and fabricated transmission parts for a flapping-wing micro-aerial vehicle (FW-MAV), which was fabricated by precision injection molding, and analyzed its warpage phenomena. First, a numerical simulation (Moldflow) was used to analyze the runner balance and temperature, pressure, and stress distributions of the base, gears, and linkage of the transmission structures in an FW-MAV. These data were then applied to fabricate a steel mold for an FW-MAV. Various process parameters (i.e., injection temperature, mold temperature, injection pressure, and packing time) for manufacturing transmission parts for the FW-MAV by precision injection molding were compared. The Taguchi method was employed to determine causes of warpage in the transmission parts. The experimental results revealed that the causes of warpage in the transmission parts were, in order of importance, the mold temperature, injection pressure, packing time, and injection temperature. After the transmission parts were assembled on the FW-MAV, experiments revealed that the MAV could achieve a flight time of 180 s. Mass production of the FW-MAV by precision injection molding could potentially produce substantial savings in time, manpower, and cost.

## 1. Introduction

Advancing knowledge of flapping-wing aerodynamics has prompted the development of small-scale flight vehicles. Promising studies of prototypes include Ashley [1] and a study by Pronsin et al. [2], which presented the first microelectromechanical system (MEMS)-based wing with a titanium-alloy metal (Ti_6_Al_4_V) wing structure and a parylene-C wing membrane. Yang et al. [3] fabricated a smart wing with polyvinylidene difluoride (PVDF)-parylene composite skin by an MEMS process and a four-bar linkage transmission system for flapping wings. Comparisons of flapping-wing micro-aerial vehicles (FW-MAVs) are given Table 1 [1,2,3,4,5,6,7,8,9,10,11,12,13,14,15,16,17,18,19,20,21,22,23,24,25,26,27,28,29,30].

Precision injection molding (PIM) is used to manufacture microstructures and is among the most common and versatile methods for mass-producing complex plastic parts. Sul et al. [31] numerically simulated an experimental micro-nozzle. The mold temperature was found to be a key process parameter in injection molding. Shen et al. [32] applied micro-injection molding and micro-injection compression molding to form light-guided plate microstructures. The mold temperature was found to be the key parameter in both processes. Although PIM is a very simple method, the interplay among processing conditions, mold geometry, and material properties is extremely complex. This complexity has motivated the development of process modeling software [33]. Wan et al. [34] performed a Moldflow analysis of window frame manufacturing by injection molding. Jansen et al. [35] studied the effect of processing conditions on mold shrinkage of seven common thermoplastic polymers. The holding pressure was a key parameter, while the effect of the melt temperature was slightly less important. Gao et al. [36] indicated an adaptive optimization method based on the Kriging surrogate model to minimize part warpage by optimizing process parameters for injection molding. The melt temperature was a major key factor affecting warpage of the parts. Hakimian and Sulong [37] investigated the effects of injection molding parameters and types of thermoplastic composites on the shrinkage and warpage properties through numerical simulations using an experimental design method. Oktem et al. [38] focused on applying the Taguchi optimization technique to find optimal levels of process parameters used in the injection of thin-shell plastic components by improving the warpage problem with shrinkage variations. Su et al. [39] revealed optimization of the evaluation of micro-injection molding parameters of a light-guide plate with the Taguchi method. Significant factors affecting the depth of the v-cut were the mold temperature, packing pressure, packing switching, and packing time.

The goal of this study was to use a plastic material (polyoxymethylene, POM) instead of aluminum for the transmission parts of an FW-MAV. This study applied a numerical simulation to calculate the value of the runner balance and temperature, pressure, and stress distributions of the transmission components of an FW-MAV. Following results of a numerical simulation, data were used as a reference for mold fabrication. Then, this study employed PIM to manufacture transmission parts for an FW-MAV. The processing windows of PIM for the transmission parts were discussed. The aim of this study was to apply the Taguchi method to determine the minimum warpage of transmission parts of an FW-MAV fabricated by PIM. Finally, the optimal processing method for mass producing the MAV parts was determined.

## 2. Experimental Methods

**Figure 1:**
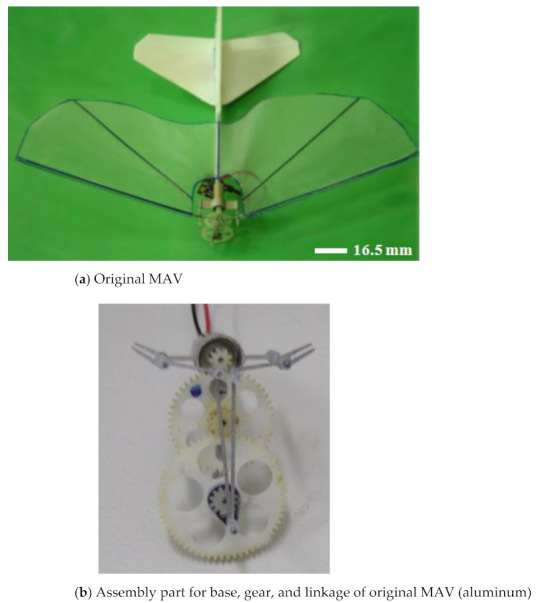
Product of the original flapping-wing MAV.

**Figure 2:**
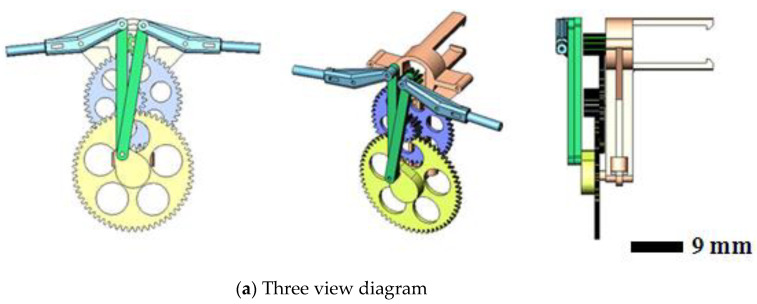

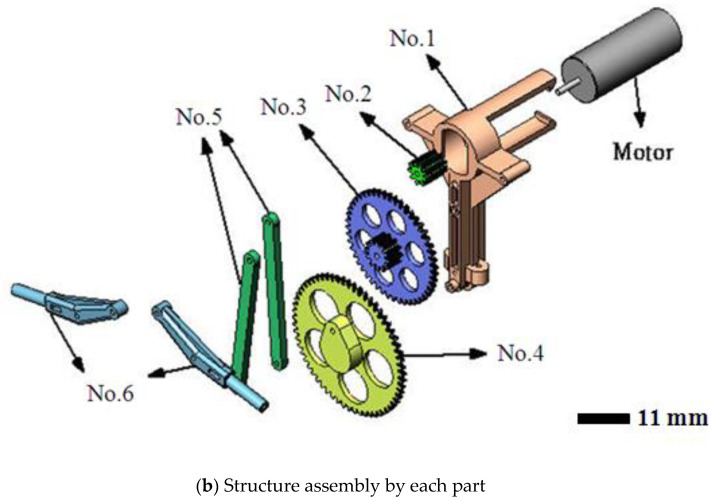

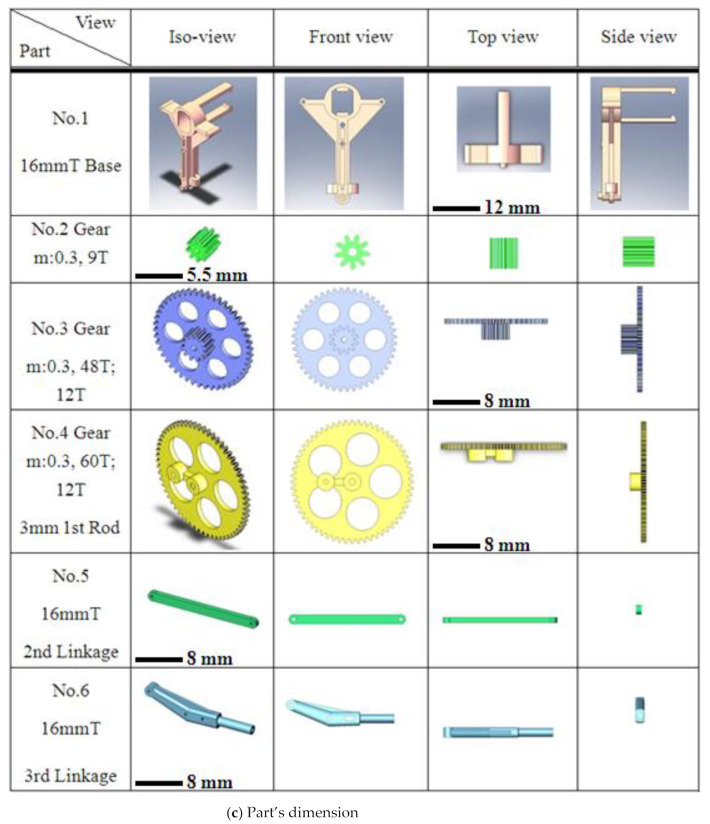
The dimensions of flapping-wing MAV’s parts.

The original MAV and its transmission parts (aluminum, Al 6061, Tripod Aluminum Co., Ltd., Changhua, Taiwan) that were developed by the authors’ lab are shown in Figure 1a,b. The transmission parts of the original MAV were fabricated by a wire computer numerical control (CNC) process, which is expensive and time consuming. The objective of this study was to find an effective alternative. This study redesigned the transmission parts (using plastic material) of an FW-MAV and employed PIM to fabricate the transmission parts. Figure 2 shows the dimensions of the transmission parts of the FW-MAV, consisting of the base, gears, and linkages.

Numerical simulation (Moldflow software, Autodesk, San Rafael, CA, USA) was used to simulate warpage of the base, gears, and linkages and to balance the runner of the FW-MAV. The governing equations for mass, momentum, and energy conservation for a non-isothermal, generalized Newtonian fluid are as follows.

Continuity equation:

(1) $$\frac{\partial \rho }{\partial t}+\frac{\partial (\rho u)}{\partial x}+\frac{\partial (\rho v)}{\partial y}+\frac{\partial (\rho w)}{\partial z}=0$$

Momentum equation:

(2) $$\rho \left(\frac{\partial u}{\partial t}+u\frac{\partial u}{\partial x}+v\frac{\partial u}{\partial y}+w\frac{\partial u}{\partial z}\right)=-\frac{\partial P}{\partial x}+\eta \left(\frac{\partial^{2}u}{\partial x^{2}}+\frac{\partial^{2}u}{\partial y^{2}}+\frac{\partial^{2}u}{\partial z^{2}}\right)+\rho g_{x}$$

(3) $$\rho \left(\frac{\partial v}{\partial t}+u\frac{\partial v}{\partial x}+v\frac{\partial v}{\partial y}+w\frac{\partial v}{\partial z}\right)=-\frac{\partial P}{\partial y}+\eta \left(\frac{\partial^{2}v}{\partial x^{2}}+\frac{\partial^{2}v}{\partial y^{2}}+\frac{\partial^{2}v}{\partial z^{2}}\right)+\rho g_{y})$$

(4) $$\rho \left(\frac{\partial w}{\partial t}+u\frac{\partial w}{\partial x}+v\frac{\partial w}{\partial y}+w\frac{\partial w}{\partial z}\right)=-\frac{\partial P}{\partial z}+\eta \left(\frac{\partial^{2}w}{\partial x^{2}}+\frac{\partial^{2}w}{\partial y^{2}}+\frac{\partial^{2}w}{\partial z^{2}}\right)+\rho g_{z}$$

Energy equation:

(5) $$\rho c_{p}\left(\frac{\partial T}{\partial t}+u\frac{\partial T}{\partial x}+v\frac{\partial T}{\partial y}+w\frac{\partial T}{\partial z}\right)=k\left(\frac{\partial^{2}T}{\partial x^{2}}+\frac{\partial^{2}T}{\partial y^{2}}+\frac{\partial^{2}T}{\partial z^{2}}\right)+\eta {\dot{\gamma }}^{2}$$

Then,

(6) $$\dot{\gamma }=\sqrt{{\left(\frac{\partial u}{\partial x}\right)}^{2}+{\left(\frac{\partial v}{\partial y}\right)}^{2}+{\left(\frac{\partial w}{\partial z}\right)}^{2}}$$

where *x*, *y*, and *z* are Cartesian coordinates, t is the time, ρ is the density, *u*, *v*, and *w* are velocities, *P* is pressure, *g* is gravity, η is viscosity, *c_p_* is the specific heat, *k* is the thermal conductivity, *T* is the temperature, and $\dot{\gamma }$ is the shear rate.

The model for fluid viscosity is

(7) $$\eta (\dot{\gamma },T,P)=\frac{\eta_{0}(T,P)}{1+{\left(\frac{\eta_{0}\dot{\gamma }}{\tau^{*}}\right)}^{1-m}}$$

(8) $$\eta_{0}(T,P)=D_{1}\exp\left[-\frac{A_{1}\left(T-T*\right)}{A_{2}+\left(T-T*\right)}\right]\;,\;T<T^{*}$$

(9) $$T*\left(P\right)=D_{2}+D_{3}P$$

(10) $$A_{2}={\tilde{A}}_{2}+D_{3}P$$

(11) $$\eta_{0}(T,P)=\infty ,\;T>T*$$

where T* denotes the glass-transition temperature and *m* represents the flow index.

Boundary and initial conditions were as follows:

(12) $$u=v=w=0;\;\begin{array}{c} T=T_{w};\; \end{array}\frac{\partial P}{\partial n}=0\;\mathrm{at}z=\pm h\;(\mathrm{on}\;\mathrm{the}\;\mathrm{mold}\;\mathrm{wall})$$

(13) $$\frac{\partial u}{\partial z}=\frac{\partial v}{\partial z}=\frac{\partial w}{\partial z}=\frac{\partial T}{\partial z}=0=\frac{\partial T}{\partial z}\;\mathrm{at}z=0(\mathrm{on}\;\mathrm{the}\;\mathrm{centerline})$$

(14) P=0(at the flow front)

(15) $$v=v_{e}\left(x,y,z,t\right)\;(\mathrm{at}\;\mathrm{the}\;\mathrm{inlet})$$

where *T_w_* is the mold temperature, *n* is the normal direction and *v_e_* is the inlet velocity.

The energy balance on the solid–liquid interface was calculated by the following equation:

(16) $$T_{s}=T_{l}=T_{m}\;at\;z=s\left(x,y,t\right)\;for\;t>0$$

(17) $$k_{s}\left(\frac{\partial T_{s}}{\partial n}\right)\left|{}_{z=s\left(x,y,t\right)}-\right.k_{l}\left(\frac{\partial T_{l}}{\partial n}\right)\left|{}_{z=s\left(x,y,t\right)}=\rho_{l}\right.L_{h}\frac{\partial s}{\partial t}$$

where *T_s_* is the solid temperature, *T_l_* is the liquid temperature, *T_m_* is the freezing temperature, *s* is the z-coordinate for the solid-liquid interface, *L_h_* is the latent heat, *k_s_* is solid thermal conductivity, and *k_l_* is liquid thermal conductivity.

The model contained the following: (a) five independent variables of three velocities (*u*, *v*, and *w*), one pressure (*P*), and one temperature (*T*); and (b) one dependent variable of viscosity (η).

**Figure 3:**
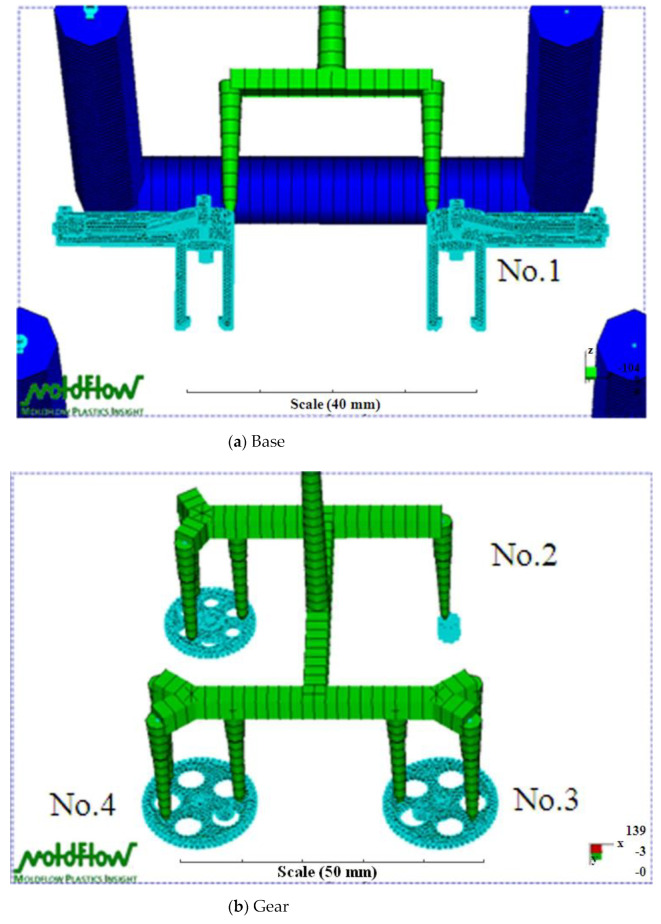

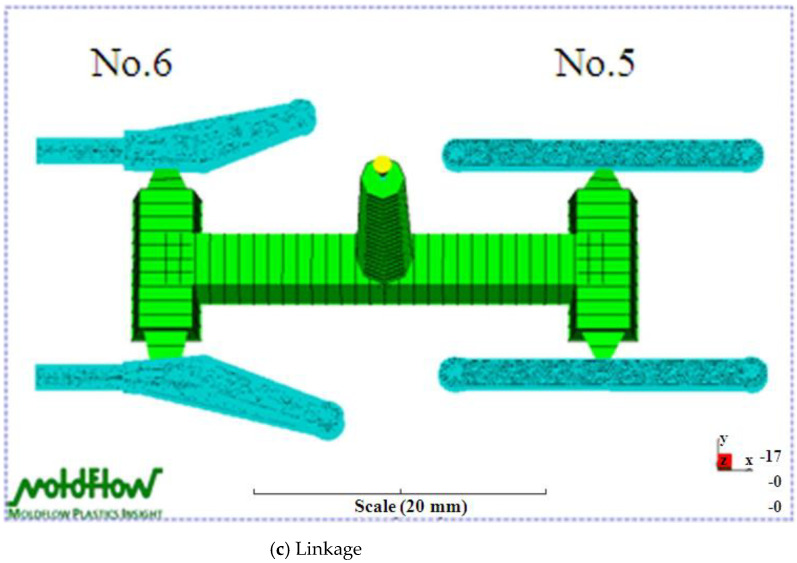
The meshes of Moldflow analysis on the parts of the flapping-wing MAV.

The governing equations in this study were solved by a finite element method. For details of the numerical simulations, see Chiang et al. [40]. A 3D mesh in the Moldflow analysis was used to examine the transmission parts (i.e., base, gears, and linkages) of the FW-MAV. The base parts of the FW-MAV model included 22,100 mesh elements, which were four-node tetrahedral elements, and 11,182 nodes (Figure 3a). The calculation time was about 2159 s for this case. The model had 19,402 mesh elements and 9800 nodes for the gear parts of the FW-MAV (Figure 3b). The calculation time was about 2608 s. Figure 3c shows that the linkage model of the FW-MAV had 32,056 mesh elements and 16,059 nodes. The calculation time was roughly 6029 s. Calculations were performed with Moldflow software (vers. 2009) on a PC equipped with a Pentium 6 3.5-GB CPU, 8 GB of memory, and a 1-TB hard drive.

**Figure 4:**
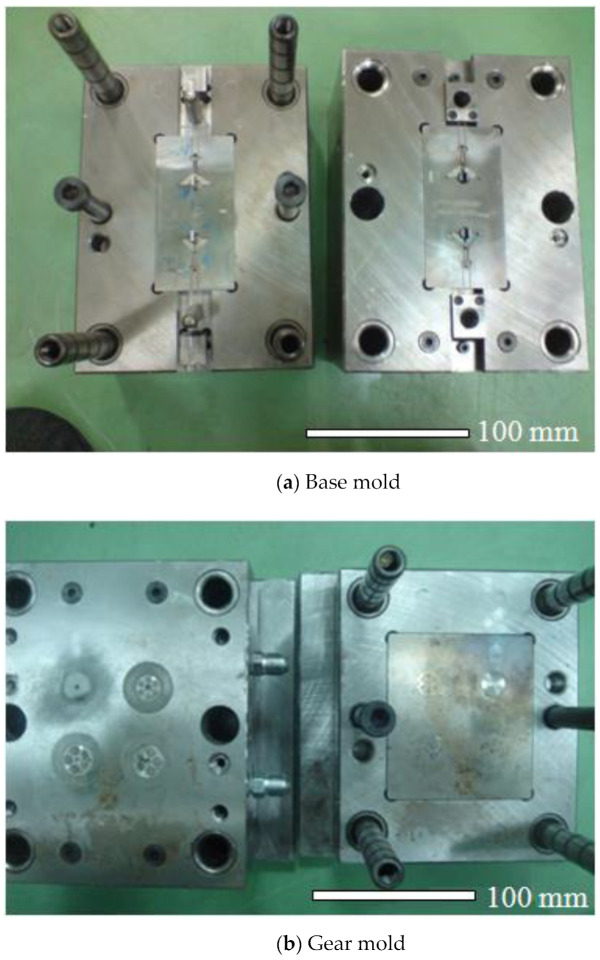

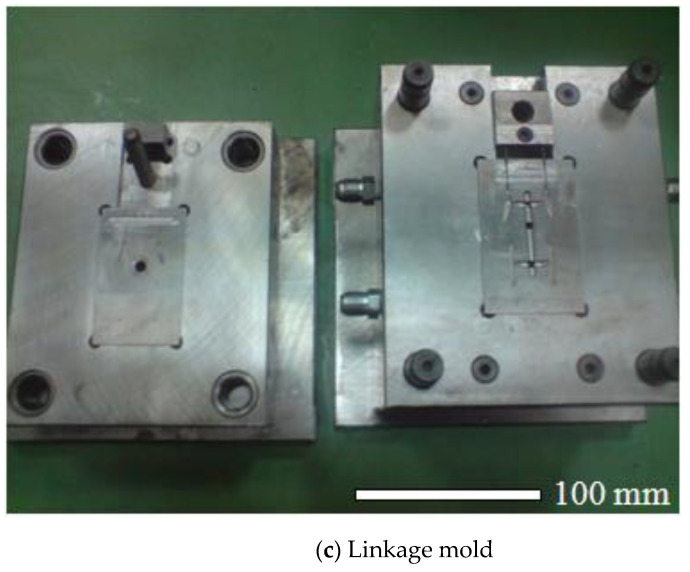
Molds for flapping-wing MAV.

A three-plate mold was utilized during PIM. The mold material was T−20. The inlet gate of the three-plate mold had a sidewall pin gate. The mold hardness was 350 micro Vickers (HMV). Figure 4 shows the mold and cavity dimensions for the base, gear, and linkage parts of the FW-MAV. The injection molding machine (220S; Arburg Co., Ltd., Loßburg, Germany) had a screw diameter of 18 mm and clamping force of 25 tons. The mold temperature control machine was the 300S type (Regloplas, St Gallen, Switzerland), with an accuracy of ±1 °C. The transmission parts of the FW-MAV used POM (Delrin^@^ 900P, DuPont, Wilmington, DE, USA). Its density, tensile strength, tensile modulus, and flexural modulus were 1.42 g/cm^3^, 70 MPa, 3.3 GPa, and 3.0 GPa, respectively. Its distortion temperature was 162 °C. POM material is a milky-white opaque crystalline thermoplastic with a high elastic modulus, high rigidity, and high hardness, and its specific strength and specific rigidity are close to that of metal. It has strong tensile strength, high flexural strength, reverse fatigue, and good impact resistance. Its friction coefficient is small, its wear resistance is good, and its dimensional stability is good. To sum up, the POM material was selected as the transmission part’s material in this study. The objective was to obtain optimal processing for the transmission parts of the FW-MAV by PIM. PIM processing parameters were the mold temperature (A), melt temperature (B), injection pressure (C), and packing pressure (D). Table 2 indicates the levels and values of the processing parameters of PIM using POM. Due to the numerous and widely varying process parameters involved, the Taguchi [41] method was used to optimize processing conditions in order to minimize warpage of the transmission parts of the FW-MAV. Determining the relative significance of these four process parameters required an array of experiments with as many as 3^4^ runs each. An L_9_ orthogonal array table was generated to determine the optimal processing conditions to produce minimum warpage during PIM.

**Figure 5:**
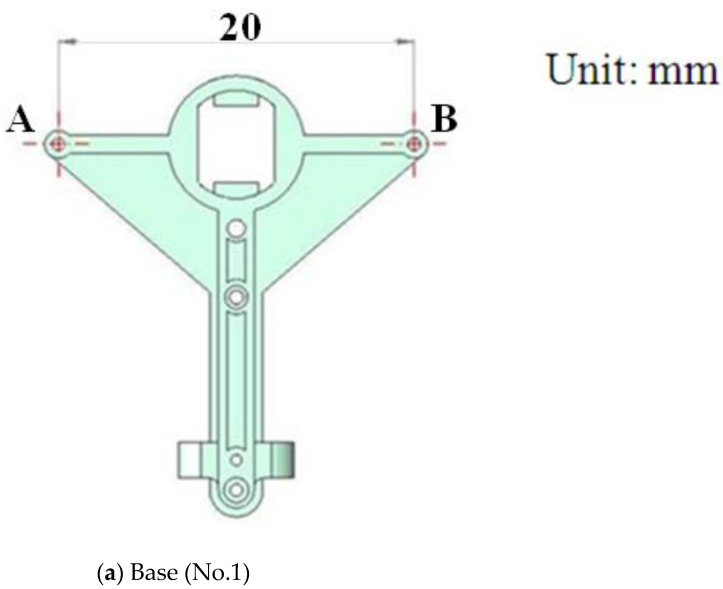

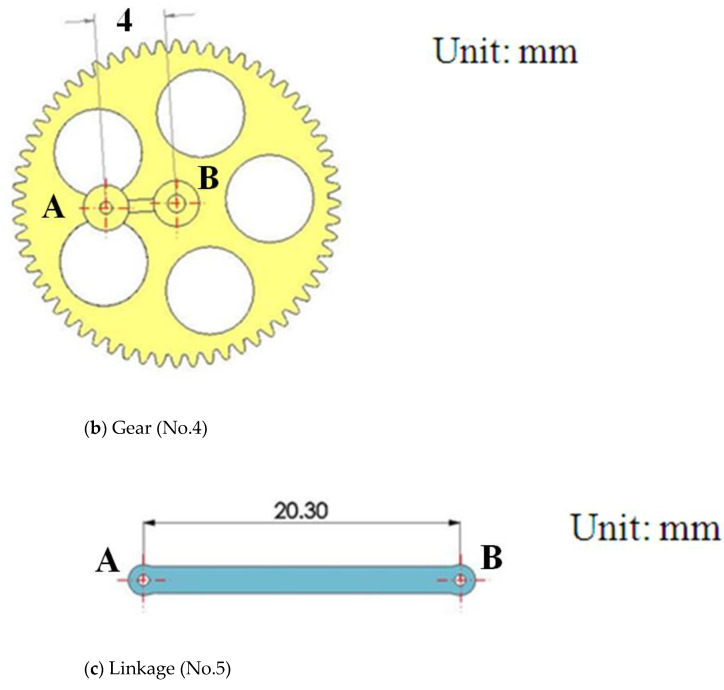
The measurement points for each part of the flapping-wing MAV.

The error allowance was essential during the final assembly of the MAV after PIM was used to fabricate each part. The main objective of this study was to analyze the warpage of the transmission parts. This study optimized process conditions to find relationships between process parameters and warpage for each transmission part of the FW-MAV. A coordinate measurement machine (CMM) was used to measure the product error of each part of the FW-MAV. This CMM had a three-dimensional (3D) laser scanner (Hawk, Nextc, UK), which measured the warpage of the transmission parts. Figure 5 reveals the measurement points used to calculate the warpage of each transmission part of the FW-MAV.

An analysis of variance (ANOVA) was separately performed for the warpage results of each transmission part of the FW-MAV, over the noise performance measure (NPM) of the signal-to-noise (S/N) ratio. The sum of squares of the total variance vector, $SS_{Total},\;$and total degrees of freedom, $DOF_{Total}$, were calculated using the following equations:

(18) $$SS_{Total}=\left[\sum_{i=1}^{n}\sum_{j=1}^{r}y_{ij}{}^{2}\right]-n\times r\times {\bar{y}}^{2}\;\mathrm{and}$$

(19) $$DOF_{Total}=n\times r-1$$

The sum of squares of the factor effect vector $SS_{Factor}\;$and factor degrees of freedom $DOF_{Factor}$ were calculated using the following equations.

(20) $$SS_{Factor}=\frac{n\times r}{L}\overset{L}{\underset{k=1}{\sum }}{\left({\bar{y}}_{k}-\bar{y}\right)}^{2}$$

(21) $$DOF_{Factor}=L-1$$

The sum of squares of the error vector,$\;SS_{Error},$ and error degrees of freedom, $DOF_{Error}$, were calculated with the following equations.

(22) $$SS_{Error}=\sum_{i=1}^{n}S_{i}{}^{2}\times \left(r-1\right)\;\mathrm{and}$$

(23) $$DOF_{Error}=n\times \left(r-1\right)$$

The calculated experimental error, S, was

(24) $$S=\sqrt{\frac{SS_{Error}}{DOF_{Error}}}$$

In these equations, *y_ij_* is the data from the j-th replicate of the i-th experiment, *ȳ_k_* is the response of the factor at the k level, *S_i_* is the standard deviation per experiment, *n* is the experiment, *r* is the number of replicates, and *L* is the level number.

## 3. Results and Discussion

**Figure 6:**
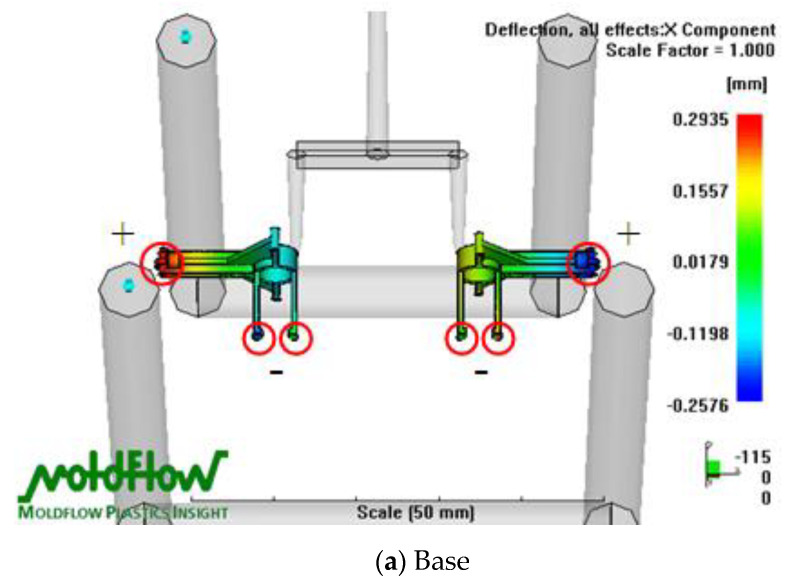

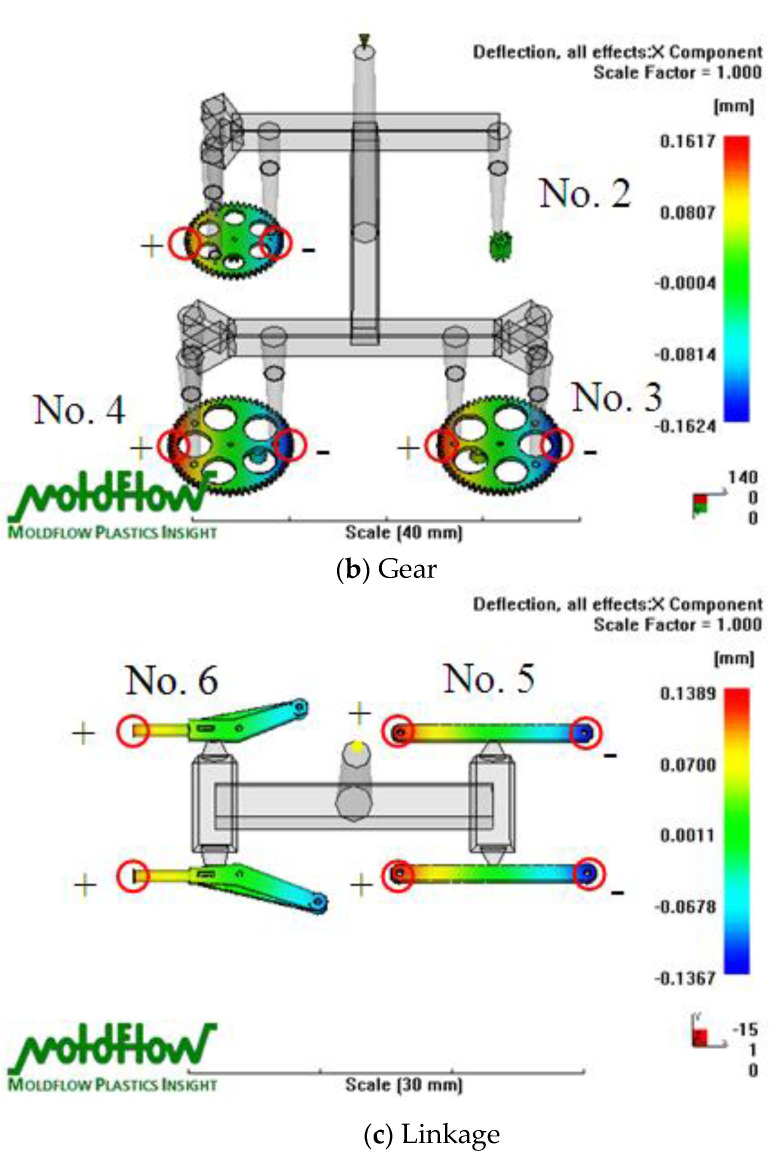
Deflection distribution of X-Z plane for Moldflow analysis on the flapping-wing MAV’s parts. The red circle is the end point of the plastic flow front flowing into the mold cavity.

Figure 6 indicates the deflection distribution in the X-Z plane of the transmission parts of the FW-MAV in the Moldflow analysis. The deflection analytical results (Figure 6a) show that the maximum negative (−) deflection was at the base end close to the motor, whereas the maximum positive (+) deflection was at the other end of the base. The maximum warpage value was 0.2935 mm for the deflection difference of the base part. It could be seen from the results that the amount of warpage was large, so it was necessary to adjust the characteristic size of the mold insert part during the mold making process to improve the accuracy of the finished product (base). The maximum deflection was positive on the left side of the gear (Nos. 3 and 4) of the FW-MAV and negative on the right side (Figure 6b). The maximum warpage value was 0.1617 mm for the deflection difference of the gear part. The maximum warpage was mostly concentrated in the large gear and middle gear. The results showed that the warpage caused the outer diameter of the gear to shrink and create errors in the fit between the gears. The warpage caused by the uneven shrinkage of the gear could be avoided by increasing the characteristic size of the mold cavity to improve the accuracy of the finished product (gear) during the manufacture of the mold. Figure 6c reveals that the positive deflection was at its maximum on the left end of the linkage (Nos. 5 and 6), and the negative deflection was maximum on the right end of the No. 5 linkage. The maximum warpage value was 0.1389 mm for the deflection difference of the linkage part. It could also be seen from the results that the amount of warpage was large; hence, it was necessary to adjust the characteristic size of the mold insert part when making the mold to improve the accuracy of the finished product (linkage).

**Figure 7:**
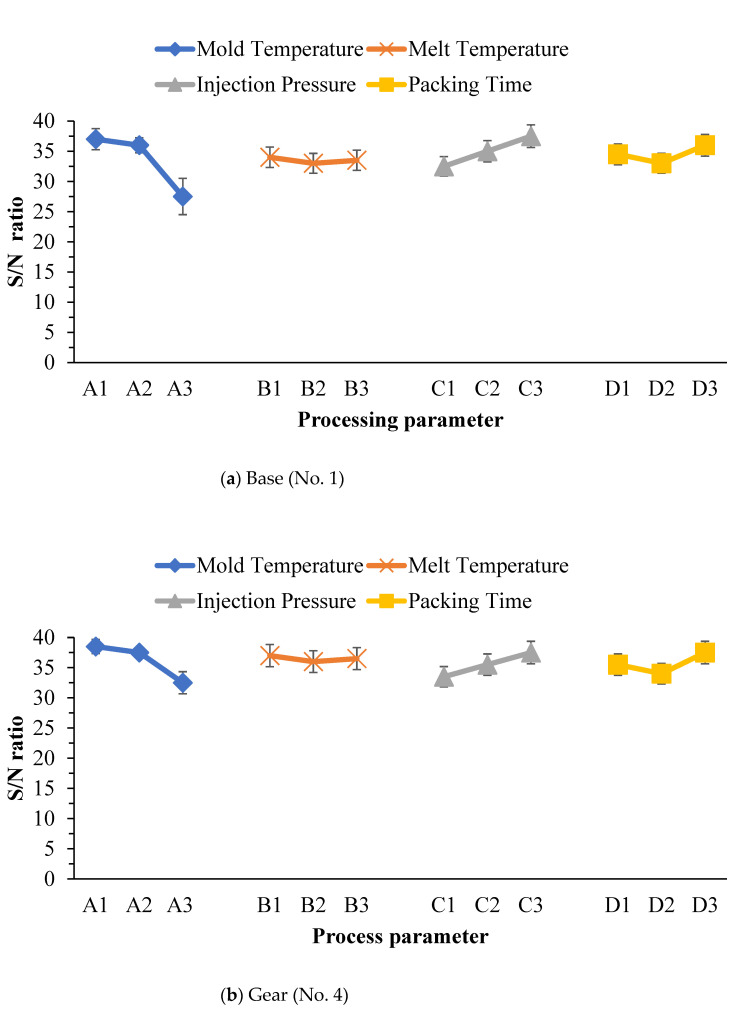

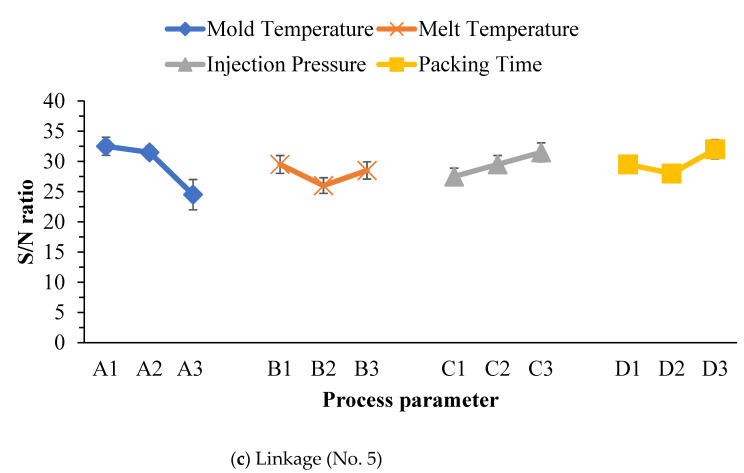
Variation of the S/N ratio with factor levels for warpage of various parts of the flapping-wing MAV.

This study used CMM to measure warpage in the transmission parts of an FW-MAV produced by PIM. The goal of optimal PIM processing is to minimize warpage; the S/N ratio of the Taguchi method must reach a minimum value. Table 3, Table 4 and Table 5 display S/N ratios of warpage of the base-gear-linkage parts of the FW-MAV. Figure 7a shows the S/N ratio with factor levels for warpage of the base part based on Table 3 in PIM. Optimal factor levels for minimizing warpage during PIM were A3B2C1D2 based on this figure’s results. These optimized factor levels meant that a mold temperature of 100 °C, a melt temperature of 210 °C, an injection pressure of 300 bar, and a packing time of 1.5 s were optimal. The data also revealed that the mold temperature was the key process parameter, followed by the injection pressure, and packing pressure while the melt temperature was inconsequential. According to the ANOVA, factor integration of the error term variation did not influence the error of the calculated results of the S/N ratio of the variation, which was large; this meant that the factor had an influence, or otherwise it would belong to the result of the experimental error. S/N ratios of warpage of the gear parts of the FW-MAV are given in Table 4. Table 5 shows the S/N ratio of warpage of the linkage part for the FW-MAV.The variance analysis table of the S/N ratio of warpage of the base part with PIM clearly showed that the variance of the mold temperature’s results was larger than those of the other three process parameters (Table 6). We integrated the factor variances of B, C, and D into the error term and obtained pooled errors, as shown in Table 6. One can see from the table that the most important parameter was the mold temperature with a confidence level of 99.76%. To sum up, we found that the mold temperature was the most important process parameter for PIM for making the base part of an FW-MAV, based on Table 3, Table 6, Table 7 and Figure 7a. 

Figure 7b reveals S/N ratios with factor levels for warpage of the gear parts fabricated by PIM. Levels of optimal factors for minimizing warpage were A3B2C1D2 during PIM. These levels of optimized factors were a mold temperature of 100 °C, a melt temperature of 210 °C, an injection pressure of 300 bar, and a packing time of 1.5 s. The mold temperature was the key process parameter for minimizing warpage of the gears, followed by the injection pressure, packing time, and melt temperature. From the ANOVA results, the change in the error term did not affect the integration of the factor, which was larger than the change in the error of the S/N ratio of the calculated results, indicating that the factor had an influence, otherwise it belonged to the experimental error. The table with results of the S/N ratio variance analysis of the warpage of PIM gear parts clearly shows that the variance of the mold temperature’s results was larger than those of the other three process parameters (Table 6). We integrated the factor variances of B, C, and D into the error term and obtained the pooled errors in Table 7. It could be seen from the table that the most important parameter at a 94.60% confidence level was the mold temperature. Finally, this study determined that the mold temperature was the most important process parameter for PIM for making the gear parts of an FW-MAV, based on Table 4, Table 6, Table 7 and Figure 7b.

Table 5 shows the S/N ratio of warpage of the linkage part for the FW-MAV. Figure 7c indicates the S/N ratio with factor levels for warpage of the linkage part with PIM. Levels of optimum factors that were statistically likely to minimize warpage during PIM were A3B2C1D2. These optimized factors were a mold temperature of 100 °C, a melt temperature of 210 °C, an injection pressure of 300 bar, and a packing pressure of 1.5 s. The data revealed that the mold temperature was the key process parameter for minimizing warpage of the linkage part, followed by the injection pressure, packing time, and finally the melt temperature. By way of the ANOVA, transformation of the error term did not affect integration of the factor, which was larger than the transformation of the error S/N ratio of the calculated outcome, indicating that the factor had an influence, otherwise it belonged to the experimental error. The table with the results of the S/N ratio variance analysis of warpage of the linkage part in PIM clearly shows that the variance of the mold temperature’s results was larger than those of the other three process parameters (Table 6). We integrated the factor variances of B, C, and D into the error term. We obtained pooled errors in Table 7, which showed that the most important parameter at a 91.25% confidence level was the mold temperature. Finally, this study determined that the mold temperature was the most important processing parameter for PIM for making the linkage part of an FW-MAV, based on Table 5, Table 6, Table 7 and Figure 7c.

To sum up, the mold temperature was the most important processing parameter for PIM of the transmission parts of an FW-MAV.

**Figure 8:**
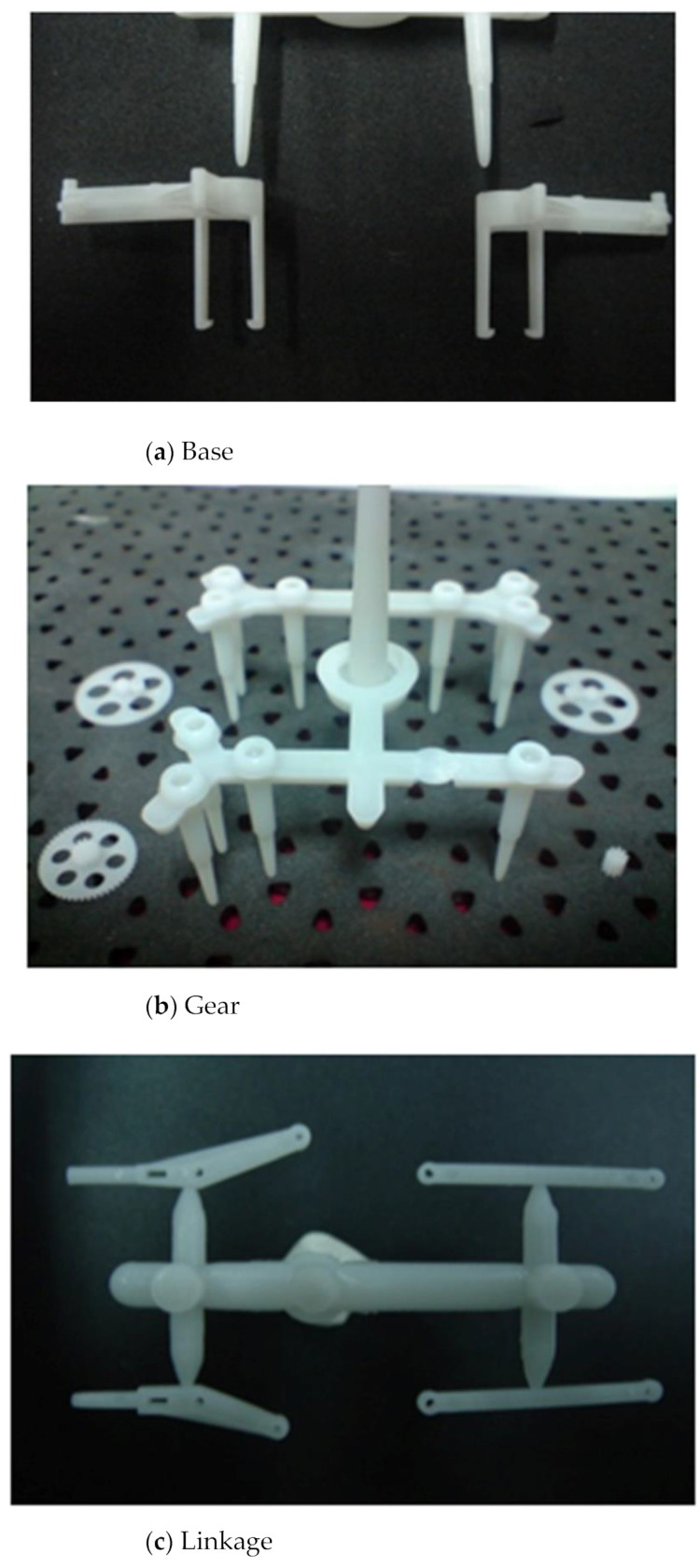

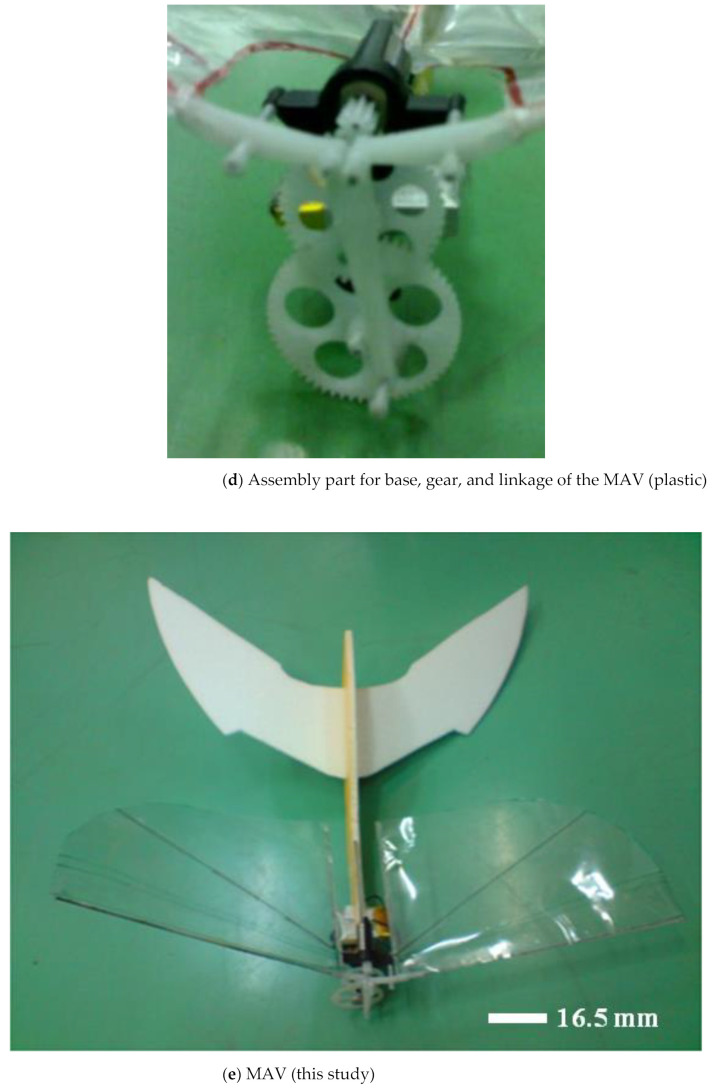
The real product for transmission structures of the flapping-wing MAV.

Figure 8 shows the various molded parts of the transmission structure by PIM; the authors assembled these components as transmission parts on the FW-MAV. This study was able to fabricate good parts and assemble a fabricated FW-MAV.

Table 8 compares the various properties of the FW-MAV with transmission components made of aluminum and plastic materials. The plastic components were lighter than the aluminum components. The weight of the transmission parts of the FW-MAV could be reduced by about 10% using plastic materials. Compared to aluminum parts, when plastic parts were used to mass produce FW-MAV transmission parts, the production process was simpler, the reproduction rate was higher, the production time was shorter, and the cost was lower. Finally, a redesigned and manufactured FW-MAV (transmission parts using plastic material) exhibited longer flight endurance.

The passive design aspect of improving the performance of the wing frames for FW-MAVs in this study tended to provide a simpler flying platform with a moderate transmission module (base, linkage and gears) but it produced bountiful and interesting phenomena that deserve further biomimetic study. The unsteady lift data and the highly enhanced thrust locomotion acquired from wind tunnel testing greatly contributed to the successful flight of our MAVs. Finally, with the advantages of being lightweight and size miniaturization, we effectively extended the existing domain of MAVs. We herein found natural animals with inclined figure-of-eight flapping similar to our MAV by empirically comparing the wingtip trajectories [42].

## 4. Conclusions

The goal of this research was to fabricate transmission parts of an FW-MAV by PIM. The objective was to optimize fabrication process parameters using minimum warpage of the transmission parts as the judgment point. This study successfully used numerical simulations to acquire data as references for mold manufacturing. We determined that the mold temperature was the key process parameter of any transmission part of the FW-MAV during PIM. The optimal factor levels in terms of minimizing warpage during PIM were predicted to be A3B2C1D2 for all parts (base, gears, and linkage) of the FW-MAV. The optimal values were a mold temperature of 100 °C, a melt temperature of 210 °C, an injection pressure of 300 bar, and a packing time of 1.5 s. Finally, the experimental results of this study were successfully used to fabricate the transmission parts of an FW-MAV by PIM. The redesigned and fabricated MAV enabled not only the mass production by PIM, but also increased the flight endurance.

## Figures and Tables

**Table 1:** References for flapping-wing micro-aerial vehicles.

Author	Material	Flapping-Wing Type	Fabrication	Flight Time
Ashley [1]	Polymer	Fixed wing	Unknown	960 s
Sirirak [2]	Titanium alloy /carbon fiber	Bird	Microelectromechinal system (MEMS)	80 s
Yang [3]	Polyvinylidene dufluonicle (PVDF)	Bird	MEMS	90 s
Ramasamy [4]	Composite carbon fiber	Bird	MEMS	Angle degree
Zhang [5]	Carbon fiber reinforced polymer	Bird	MEMS	100 s
Sai [6]	Non-woven fabric plastic	Bird	MEMS	Angle degree
Deng [7]	Polymer	Bird	Performed by author’s Lab	Angle degree
Phan [8]	Carbon/epoxy	Bee	Performed by author’s Lab	Angle degree
Phan [9]	Carbo/epoxy	Bee	Performed by author’s Lab	Flapping Moment
Phan [10]	Polyethylene terephthalate	Beetle	MEMS	1.5 m height on 3 s
Truong [11]	POM	Bird	CNC processing	Optimization for gear
Badrya [12]	Simulation	Dipteral insert	Simulation	Flapping force
Hassanalian [13]	Polymer	Bird	Performed by author’s Lab	50 m height
Nguyen [14]	POM/carbon fiber	Bird	CNC	Thrust
Bluman [15]	Model/simulation	Bumblebee	Model/simulation	Angle degree
Lu [16]	Carbon fiber	Bat	Performed by author’s Lab	Nosie
Herrero [17]	Polystyreme	Bird	CNC	Angle degree
Yang [18]	POM/Al/Ti	Bird (dove)	Bird	CNC
Nan [19] (review)	Polymer Cabon fiber Ti	Insect Bird	MEMS CNC Mold	Thrust Angle degree
Nguyen [20]	POM/Carbon fiber	Insect	CNC	Flight speed
Jankauski [21]	Model/simulation	Insect	Model/simulation	Frequency
Badrya [22]	Polymer	Insect	CNC	Lift force drag force
Cao [23]	Polyacrylate	Insect	CNC	Flight speed
Gallar [24]	Carbon fiber	Fiber	CNC	Flight speed
Lane [25]	PLA/carbon spars/epoxy/Malar	Insect (bumblebee)	3D printing	Thrust
Lee [26]	Model/simulation	Bird	Model/simulation	Angle degree
Dong [27]	Polymer	Bird	Performed by author’s Lab	Angle degree
Yoon [28]	Carbon/epoxy	Bird	CNC	Camber angle
Nauyen [29]	Model/simulation	Insect (beetle)	Model/simulation	Angle of attack
Wang [30]	Model/simulation	Insect	Model/simulation	Angle degree
This study	POM	Bird	CNC/mold	180 s

**Table 2:** Processing parameters for base/gear/linkage.

Parameters	Levels		
	Level 1	Level 2	Level 3
A. Mold temperature (°C)	80/80/80	90/90/90	100/100/100
B. Melt temperature (°C)	200/200/200	210/210/210	220/220/220
C. Injection pressure (bar)	300/300/300	400/400/400	500/500/500
D. Packing time (s)	1/1/1	1.5/1.5/1.5	2/2/2

**Table 3:** S/N ratio of warpage of base part for the MAV.

Exp.	*y_1_*	*y_2_*	*y_3_*	*y_4_*	*y_5_*	Ave.	*S*	*S/N*
1	0.0183	0.0182	0.0178	0.0179	0.0175	0.0179	0.0003	35.0238
2	0.0172	0.0172	0.0162	0.0165	0.0160	0.0166	0.0006	35.7912
3	0.0118	0.0120	0.0110	0.0120	0.0100	0.0114	0.0009	39.1483
4	0.0124	0.0124	0.0126	0.0124	0.0127	0.0125	0.0001	38.0152
5	0.0132	0.0132	0.0136	0.0137	0.0138	0.0135	0.0003	37.2654
6	0.0185	0.0185	0.0190	0.0192	0.0189	0.0188	0.0003	34.4095
7	0.0320	0.0320	0.0320	0.0320	0.0320	0.0320	0.0000	29.8970
8	0.0400	0.0450	0.0420	0.0430	0.0440	0.0428	0.0019	27.3291
9	0.0370	0.0370	0.0360	0.0380	0.0400	0.0376	0.0015	28.3963
Optimum	0.001	0.002	0.001	0.003	0.001	0.0022	0.0000	53.1515
Average						0.0226	0.0007	33.9195

**Table 4:** S/N ratio of warpage of gear part for the MAV.

Exp.	*y_1_*	*y_2_*	*y_3_*	*y_4_*	*y_5_*	Ave.	*S*	*S/N*
1	0.017	0.016	0.015	0.014	0.016	0.0156	0.0011	36.4653
2	0.012	0.010	0.014	0.016	0.014	0.0132	0.0023	36.6555
3	0.010	0.005	0.007	0.006	0.009	0.0074	0.0021	42.5701
4	0.012	0.014	0.011	0.010	0.009	0.0112	0.0019	39.9711
5	0.014	0.016	0.012	0.012	0.010	0.0128	0.0023	38.8829
6	0.019	0.019	0.019	0.019	0.019	0.0194	0.0000	34.2364
7	0.021	0.019	0.019	0.022	0.020	0.0202	0.0013	33.8195
8	0.024	0.028	0.025	0.021	0.027	0.0250	0.0027	32.2306
9	0.020	0.019	0.022	0.024	0.023	0.0216	0.0021	32.7600
Optimum	0.007	0.006	0.006	0.005	0.004	0.0056	0.0011	45.9063
Average						0.0163	0.0018	36.3990

**Table 5:** S/N ratio of shrinkage of linkage part for the MAV.

Exp.	*y_1_*	*y_2_*	*y_3_*	*y_4_*	*y_5_*	Ave.	*S*	*S/N*
1	0.036	0.037	0.035	0.036	0.037	0.0362	0.0008	28.8717
2	0.026	0.028	0.032	0.029	0.030	0.0290	0.0022	30.3543
3	0.013	0.017	0.014	0.016	0.015	0.0150	0.0016	36.4653
4	0.019	0.020	0.019	0.020	0.021	0.0198	0.0008	33.9722
5	0.023	0.022	0.025	0.026	0.028	0.0248	0.0024	31.5802
6	0.049	0.043	0.040	0.041	0.039	0.0424	0.0040	27.9570
7	0.048	0.052	0.050	0.048	0.049	0.0494	0.0017	26.1949
8	0.055	0.052	0.058	0.063	0.063	0.0582	0.0049	24.2397
9	0.054	0.052	0.059	0.061	0.060	0.0572	0.0040	24.4362
Optimum	0.010	0.011	0.008	0.009	0.007	0.0090	0.0016	41.8932
Average						0.0369	0.0025	29.3413

**Table 6:** Variance analysis of S/N ratio of base (b), gear (g), and linkage (l) parts for the MAV.

Factor	Square Sum	Degree of Freedom	Variance	F Distribution	Confidence
A	130.20 (b) 55.07 (g) 87.30 (l)	2 (b) 2 (g) 2 (l)	65.100 (b) 27.536 (g) 43.648(l)	3.4630 (b) 2.2244 (g) 2.4878 (l)	91.75% (b) 82.95% (g) 85.55% (l)
B	1.10 (b) 1.10 (g) 1.72 (l)	2 (b) 2 (g) 2 (l)	0.552 (b) 0.550 (g) 0.858 (l)	0.0293 (b) 0.0444 (g) 0.0489 (l)	2.88% (b) 4.32% (g) 4.74% (l)
C	15.29 (b) 25.40 (g) 29.19 (l)	2 (b) 2 (g) 2 (l)	7.647 (b) 12.699 (g) 14.595 (l)	0.4068 (b) 1.0259 (g) 0.8319 (l)	32.12% (b) 59.88% (g) 53.03% (l)
D	3.79 (b) 17.46 (g) 22.16 (l)	2 (b) 2 (g) 2 (l)	1.897 (b) 8.731 (g) 11.079 (l)	0.1009 (b) 0.7053 (g) 0.6315 (l)	9.49% (b) 47.77% (g) 44.36% (l)
Total	150.39 (b) 99.03 (g) 140.36 (l)	8 (b) 8 (g) 8 (l)			

**Table 7:** Pooling of errors of S/N ratio of base, gear, and linkage parts for the MAV.

Factor	Square Sum	Degree of Freedom	Variance	F Distribution	Probability	Confidence
A	130.20 (b) 55.07 (g) 87.295 (l)	2 (b) 2 (g) 2 (l)	65.100 (b) 27.536 (g) 43.648 (l)	19.3447 (b) 3.7584 (g) 4.935 (l)	0.24% (b) 8.75% (g) 5.40% (l)	99.76% (b) 91.25% (g) 94.60% (l)
B	Pooling of errors					
C	Pooling of errors					
D	Pooling of errors					
Error	20.1917 (b) 43.9594 (g) 53.064 (l)	6 (b) 6 (g) 6 (l)	3.365 (b) 7.327 (g) 8.844 (l)	S exp. Error = 1.8345 dB (b) S exp. Error = 2.7068 dB (g) S exp. Error = 2.974 dB (l)		
Total	150.3925 (b) 99.0321 (g) 140.359 (l)	8 (b) 8 (g) 8 (l)				

**Table 8:** Comparison results of the transmission structure of the flapping-wing MAV between aluminum alloy material and plastic material.

	Material Name	
	Aluminum (6061)	Plastic (POM)
Total weight (g)	2.528	2.279
Fabrication method	WCNC	Precision injection molding
Processing	Half automation	Automation
Processing flowchart	Complex	Simple
Molding time (min/group)	30	2
Reproduction	Low	High
Percent defectives	High	Low
Costs (NT dollar)	620	12.2
Assembly learning time	Long	Short
Flying time (s)	47	106

## Data Availability

The data presented in this study are available on request from the corresponding author.
